# Investigation of Photoluminescence and Optoelectronics Properties of Transition Metal-Doped ZnO Thin Films

**DOI:** 10.3390/molecules28247963

**Published:** 2023-12-06

**Authors:** Mohsin Khan, Ghazi Aman Nowsherwan, Rashid Ali, Muqarrab Ahmed, Nadia Anwar, Saira Riaz, Aroosa Farooq, Syed Sajjad Hussain, Shahzad Naseem, Jeong Ryeol Choi

**Affiliations:** 1 Centre of Excellence in Solid State Physics, University of the Punjab, Lahore 54590, Pakistan; 2 Department of Materials Science and Engineering, Ghulam Ishaq Khan Institute of Engineering Sciences and Technology, Topi 23640, Pakistan; 3 Department of Chemical Engineering, University of Engineering and Technology, Lahore 54890, Pakistan; 4 Department of Physics, The University of Lahore, Lahore 54000, Pakistan; 5 School of Electronic Engineering, Kyonggi University, Suwon 16227, Republic of Korea

**Keywords:** ZnO, transition metal-doped ZnO, optical properties, thin films, photoluminescence

## Abstract

Thin films of zinc oxide (ZnO) doped with transition metals have recently gained significant attention due to their potential applications in a wide range of optoelectronic devices. This study focuses on ZnO thin films doped with the transition metals Co, Fe, and Zr, exploring various aspects of their structural, morphological, optical, electrical, and photoluminescence properties. The thin films were produced using RF and DC co-sputtering techniques. The X-ray diffraction (XRD) analysis revealed that all the doped ZnO thin films exhibited a stable wurtzite crystal structure, showcasing a higher structural stability compared to the undoped ZnO, while the atomic force microscopy (AFM) imaging highlighted a distinctive granular arrangement. Energy-dispersive X-ray spectroscopy was employed to confirm the presence of transition metals in the thin films, and Fourier-transform infrared spectroscopy (FTIR) was utilized to investigate the presence of chemical bonding. The optical characterizations indicated that doping induced changes in the optical properties of the thin films. Specifically, the doped ZnO thin film’s bandgap experienced a significant reduction, decreasing from 3.34 to 3.30 eV. The photoluminescence (PL) analysis revealed distinguishable emission peaks within the optical spectrum, attributed to electronic transitions occurring between different bands or between a band and an impurity. Furthermore, the introduction of these transition metals resulted in decreased resistivity and increased conductivity, indicating their positive influence on the electrical conductivity of the thin films. This suggests potential applications in solar cells and light-emitting devices.

## 1. Introduction

Due to the unique qualities that zinc oxide (ZnO) nanostructures possess, a great emphasis is presently being placed on the investigation of the characteristics of metal oxides in the context of the broader field related to them. According to the periodic table, ZnO is a kind of binary compound that contains elements from Group II and Group VI. It exhibits a stable hexagonal wurtzite crystal structure at standard ambient temperature [1]. It is a very effective transparent conducting oxide (TCO) material, possessing a favorable bandgap of 3.37 eV at standard room temperature [2]. Additionally, the light transmittance through them within the visible spectrum is enhanced as the wavelength increases at first. However, such a transmittance at sufficiently higher wavelengths in the visible region decreases with the increase in the wavelength. [3]. It exhibits a notable excitonic binding energy of 60 meV [4,5]. It has been used in a wide range of electrical and optoelectronic applications [6], including UV detectors/photodetectors [7], photo-catalysis [8], solar cells [9], and gas sensors [10].

Transition metal-doped zinc oxide (TM-ZnO) thin films have garnered a substantial amount of interest and have been a topic of much research because of their one-of-a-kind properties and many potential applications in a variety of scientific and technical fields [11]. Zinc oxide is a semiconducting material that possesses a wide bandgap. As a result of its remarkable optical, electrical, and piezoelectric properties, zinc oxide has gained significant attention in recent years. The incorporation of transition metals into the ZnO lattice causes the formation of new functionalities, which in turn enables the modification of the material’s electrical and optical properties. Because of this, the range of possible uses for zinc oxide has been broadened [12].

Extensive research has been conducted on the incorporation of transition metals, including Mn, Co, Ni, Cu, and Fe, into thin films of ZnO [13,14]. Such research has generally been focused on improving their electrical band structure and optical qualities, intending to boost their magnetic characteristics. Dopants may be introduced into ZnO to bring about a variety of effects, some of which include the facilitation of charge transport, the induction of magnetic characteristics, and the alteration of the energy bandgap. The development of optoelectronic devices is made possible because of these structural modifications. The selection of a transition metal dopant, the amount of its concentration, and the procedure that was used for deposition are all key aspects that contribute to the determination of the features of the film and the various applications in optoelectronics devices [15].

Doping ZnO nanostructures with transition metals has become an increasingly popular method for altering ZnO nanostructures in recent years [6]. The ionic radii of Co and Fe are comparatively smaller, but the ionic radii of Zr are larger in comparison to zinc oxide (ZnO); these elements have improved electrical conductivity, optical characteristics, and photoluminescence [16]. It is predicted that the addition of transition elements into the ZnO lattice will increase the charge carrier density. These elements will serve as replacements for zinc atoms. Incorporating transition metals as dopants in ZnO has been proven in a multitude of different empirical studies to result in an improvement in the material’s photodetection capabilities. The results of the research revealed how Cu-doped ZnO nanostructures are used to increase the photosensitivity of visible light for use in photodetection applications [17]. The incorporation of zirconium into zinc oxide has increased the efficiency of both LEDs and photovoltaic devices [18]. The photodegradation effectiveness of ZnO doped with Mn^2+^ is somewhat lower than that of pure ZnO owing to changes in its absorption properties, which expedites the recombination of electron–hole pairs [19]. This is in comparison to the photodegradation efficiency of pure ZnO. According to the findings of research, the photodegradation of rhodamine B solution caused by exposure to ultraviolet light was significantly slowed down when the dopant Co^2+^ was included. This action may be related to the capacity of these Co^2+^ ions to trap and immobilize electrons and holes inside the ZnO lattice, hence preventing their recombination. This effect may also be attributable to the fact that these Co^2+^ ions are rather abundant [20]. Saadi et al. investigated the impact of Co and Fe doping on the structural and electrical properties of ZnO nanoparticles for energy storage applications [21,22]. Cobalt doping induces changes in crystal structure and morphology (from spherical to rod-shaped), and these changes make the optical bandgap narrow. The dielectric analysis indicates an increased dielectric constant and electrical conductivity. In comparison, Fe-doped ZnO nanoparticles were synthesized with varying Fe concentrations. The results showed improved AC conductivity, reduced impedance, and enhanced dielectric properties with Fe doping, highlighting the potential of Fe-doped ZnO nanoparticles for efficient electric storage applications.

Research has also been conducted on the photoluminescence (PL) characteristics of thin films of ZnO doped with transition metals, primarily driven by their promising prospects in the field of optoelectronic devices [23]. Extensive research has been conducted on thin films of ZnO doped with copper [24]. The presence of copper impurities in ZnO leads to the creation of localized energy levels inside the bandgap of the material, which in turn makes it easier for the material to emit visible photoluminescence. The major emission peak is often seen at a wavelength range that falls between 600 and 700 nm. This corresponds to the spectral band that is linked with orange-red light [25]. Doping with cobalt can cause a wide spectrum of PL emissions over the whole visible and near-infrared spectral regions of the spectrum. The fact that co-doped ZnO displays emission peaks mostly in the blue and green spectral regions makes it an excellent candidate for use in a variety of display and sensor applications [26]. The inclusion of manganese as a doping element in zinc oxide causes the formation of new energy levels inside the bandgap of the material. After some time has passed, this event will ultimately result in the emission of visible PL. Manganese-doped ZnO thin films can produce emissions in the green, yellow, or red portions of the electromagnetic spectrum. The precise emission color is reliant upon the percentage of manganese (Mn) present in the films, as stated in Ref. [27]. It has been observed that the incorporation of nickel as a dopant in ZnO thin films results in the production of various emissions in the visible and near-infrared areas of the electromagnetic spectrum. The nickel concentration and the precise conditions under which annealing takes place both have a role in determining the locations of the emission peaks that are seen [28]. It has been shown that the presence of iron-doped ZnO thin films results in PL emissions that are located within the visible spectrum [29].

Transition metal-doped zinc oxide (ZnO) thin films may be deposited using many processes, such as physical vapor deposition techniques like pulsed laser deposition and radio frequency (RF) magnetron sputtering [30,31,32] and chemical vapor deposition [26,29]. These methods are widely used in the field of thin-film fabrication.

This research delves into the deposition of thin films, specifically ZnO and transition metal-doped ZnO, using RF magnetron co-sputtering. This comprehensive investigation aims to unveil how the introduction of transition metal dopants (Co, Fe, and Zr) influences the structural, optical, and electrical properties of these films. This study is motivated by the desire to understand the unique characteristics induced by doping and to pinpoint the potential applications of such doped ZnO films, particularly in the realms of electrodes and electron transport layers for highly efficient solar cells. The overarching objective is to contribute to the progress of sustainable and cutting-edge energy technologies, fostering the development of more efficient and environmentally friendly solar energy devices.

## 2. Materials and Methods

### 2.1. Preparation of Thin Films

**Figure 1:**
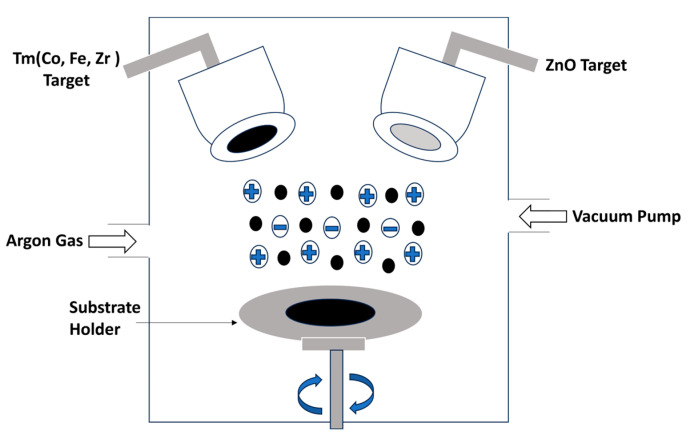
Experimental setup of co-sputtering.

Thin films of ZnO doped with transition metals (TMs) such as Co, Fe, and Zr were deposited using dual magnetron sputtering equipment, as illustrated in Figure 1. The co-sputtering process of ZnO and TMs was successfully carried out utilizing a sputtering system that combines radio frequency (RF) and direct current (DC) sputtering. The ZnO and TM targets used in this process had a purity level of 99.9% and 99.95%, respectively. Both targets had a thickness and diameter of 3 mm and 2 inches, respectively. For consistent comparison and characterization, glass was employed as a substrate in the sputtering process. Before deposition, the substrate underwent a meticulous cleansing process involving the use of acetone and isopropyl alcohol (IPA). Subsequently, nitrogen gas flow was applied to the slide to eliminate any remaining impurities and adhesive particles. The substrate was then carefully positioned onto the substrate holder, maintaining 60 mm between the substrate and the target. The sputtering process of TMs was successfully executed using DC sputtering. A DC power of 25 W was supplied, resulting in a current of 70 mA and a voltage of 275 V throughout the deposition process. The base pressure was maintained at 6.6 × 10^−3^ Pa, and the rate of substrate movement was recorded at 100 rpm.

Simultaneously, the RF sputtering technique was employed for depositing ZnO. The operational conditions for RF sputtering included a working pressure of 2.4 Pa, an RF power source of 100 W, and an operating voltage of 100 V. The flow rate of argon gas was measured at 80 sccm. The deposition time for TMs was 2 min, while ZnO required 15 min. At the commencement of the deposition process, both the ZnO and TM targets were initiated for deposition. After thirty seconds, the TM target was deactivated. Subsequently, at intervals of five minutes, the TM material was deposited for thirty seconds. The estimated thickness of the deposited thin films ranged from 250 to 270 nm, as determined by the thickness monitor operating based on the Z-factor of the target.

### 2.2. Characterization of Thin Films

During our characterization, we meticulously analyzed the structure, elemental composition, chemical bonding, optical properties, photoluminescence activity, and electrical characteristics. This comprehensive investigation relied on a diverse array of spectroscopic and microscopy techniques, including X-ray diffraction (XRD), scanning electron microscopy (SEM), Fourier-transform infrared spectroscopy (FTIR), ultraviolet-visible spectroscopy (UV-vis), photoluminescence (PL) spectroscopy, and four-probe point measurements. For X-ray diffraction data analysis, we employed a Cu K_α_ radiation source with a wavelength of 1.5406 nm (40 kV/30 mA) in conjunction with the PROTO AXRD Powered Diffraction System. The Nanosurf AFM was used to investigate the surface texture of the films using an atomic force microscope in tapping mode. Elemental analysis of the deposited thin films was conducted through energy-dispersive X-ray spectroscopy (EDX). Fourier-transform infrared (FTIR) analysis, aimed at determining sample components and the presence of chemical bonding, utilizing the Thermo Scientific Nicolet IS5 instrument. To delve into optical characteristics, a Shimadzu UV-1800 spectrophotometer was employed. The photoluminescence properties of the deposited thin films were explored using an FS5 Spectrofluorometer. The investigation of electrical characteristics in the deposited thin films employed the Ossila Four-Point Probe. This amalgamation of techniques allowed for a thorough and multi-faceted examination, offering insights into the diverse aspects of the studied materials.

## 3. Results

### 3.1. Structural Analysis

**Figure 2:**
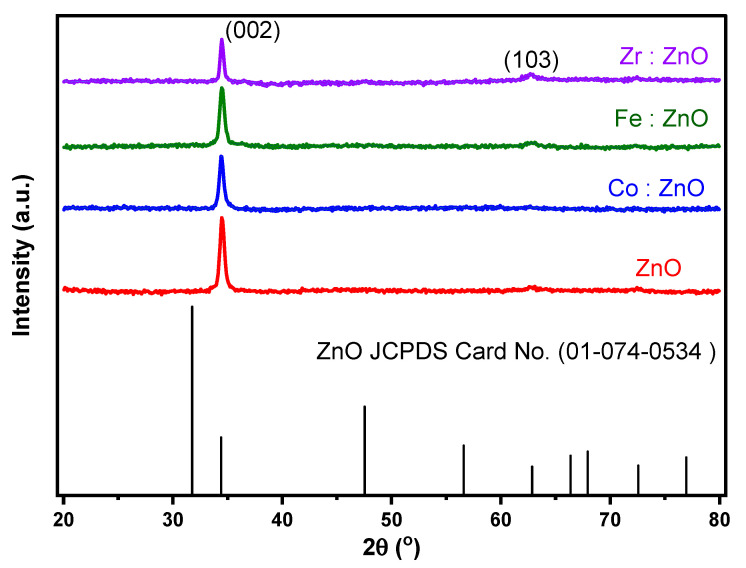
XRD of ZnO and TM-doped ZnO thin films.

The XRD pattern of ZnO and TM-doped ZnO thin films that were deposited by co-sputtering can be seen in Figure 2. The findings of the XRD analysis show that the deposits are polycrystalline and have a crystal structure that is hexagonal along the c-axis. The spectra show a peak with a significant amount of intensity that corresponds to the (002) plane of 2 at 34.5 degrees, as well as peaks that correspond to the (103) plane of 2 at 62.8 degrees. The JCPDS Card number (01-074-0534) verifies the existence of this structure. Doping the material with a variety of transition metals resulted in a reduction in the intensity of the main peak (002). The intensity peak for Fe was less affected by the reduction when compared to the peaks for Co and Zr. The XRD peak strength decreases proportionately with the degree to which this lattice distortion causes the crystal lattice to become less organized [33]. When foreign ions are introduced into the ZnO lattice, it has the potential to cause defects and disorder, which in turn lowers the intensity of the XRD peaks [34].

However, notably, discernible variations in the peak intensity, width, and position captivated our attention. The shifts in the peak intensity signaled alterations in the atomic arrangement within the crystal lattice induced by the dopants. The introduction of Fe, Co, and Zr into the ZnO structure resulted in the incorporation of foreign atoms, giving rise to new crystal planes or modifying existing ones. Consequently, the diffraction efficiency at specific angles experienced changes, leading to shifts in peak intensities. Similarly, the observed shifts in the peak position served as a clear indicator of modifications in the interplanar spacing of the ZnO crystal. Furthermore, fluctuations in the peak width were evident, reflecting changes in the crystallite size and the presence of lattice imperfections or strain introduced during the doping process. The incorporation of Fe, Co, and Zr into the ZnO thin film exerted influence over the growth and arrangement of the crystalline domains, instigating alterations in the size of individual crystallites. Additionally, the presence of dopants introduced lattice imperfections and strain, contributing to the observed broadening of the peaks.

Using the Scherrer equation, one major peak (002) and one minor peak (103) were used to determine the crystalline size (D) of the ZnO thin films that had been doped with transition metals (Co, Fe, Zr). After that, the value of (D) was rounded up to the next whole number, which was [35]

(1) $$D_{P}=\frac{k\lambda }{\beta cos\theta }$$

where k is the shape factor, λ is the X-ray wavelength, β is the full width at half maximum (FWHM), and θ is the Bragg angle of the peak with the highest intensity. The average crystallite dimensions of the transition metal-doped ZnO thin films are shown in Table 1. The doping with the TMs resulted in a reduction in the crystallite size. The ionic radius of the transition metal dopants is different from that of the zinc and oxygen ions. These dopants may not fit exactly in the ZnO lattice, which might cause strain in the material. This strain may cause damage to the crystalline structure and stop the growth of larger crystallites. As a result, the size of the crystallites diminishes [36].

The microstrain ε of the thin film is measured using the Stokes–Wilson relation, which is [37]

(2) $$\varepsilon =\frac{\beta }{4tan\theta }$$

The microstrain increased with the doping of Co, Fe, and Zr. [38].

The dislocation density increases by doping with the following sequence: Co, Fe, and Zr, as indicated in Table 1. ZnO lattice vacancies result from crystallite size decrease and strain increase.

The quantity of defects in the ZnO that have been doped with transition metal ions is represented by the dislocation density (δ). The dislocation density was found by using the following formula [39]:

(3) $$\delta =\frac{1}{D^{2}}$$

The bond length (*L*) was also found using the XRD spectra via an evaluation using the relation

(4) $$L=\sqrt{\frac{a^{2}}{3-{\left(\frac{1}{2}-u\right)}^{2}c^{2}}}$$

The internal relaxation parameter in the wurtzite structure is measured by [40].

(5) $$u=\frac{1}{3\left(\frac{a^{2}}{c^{2}}\right)+0.25}$$

The feature of a material known as its surface area calculates the entire contact area of the crystals per unit of mass. Its evaluation is crucial to maximizing the adsorption catalysis and reactions on thin film surfaces. The surface area was calculated using the Sauter relation of the form [41]

(6) $$S=\frac{{6\times 10}^{3}}{Dp}$$

where S is the specific surface area, *D* is the crystallite size, and ρ is density [42]. In this case, the density of the bulk ZnO is 5.606 gcm^−3^.

The XRD analysis revealed that the average grain size of ZnO, Co: ZnO, Fe: ZnO, and Zr: ZnO were calculated as 49.26 nm, 47.58 nm, 44.83 nm, and 43.71 nm, respectively. The corresponding microstrain of ZnO, Co: ZnO, Fe: ZnO, and Zr: ZnO were estimated as 2.95 × 10^−3^, 3.63 × 10^−3^, 3.71 × 10^−3^, and 4.02 × 10^−3^. The variations in the grain size following the introduction of zirconium, cobalt, and iron into ZnO can be ascribed to the distinct influences of these dopants which are exerted on the material’s microstructure. Transition metals (TMs) are recognized for their role as either grain growth inhibitors or restrictors in certain materials. In the context of ZnO, the incorporation of these dopants can impede grain growth, resulting in smaller and more dispersed grain sizes. The reduction in the grain size holds significance as it can offer advantages in specific applications. Smaller grain sizes contribute to improved mechanical properties, an increased surface area, and enhanced material homogeneity. It is crucial to emphasize that the behavior of dopants in materials is intricate and contingent upon factors such as the specific doping concentrations, processing conditions, and the intricate interactions between dopants and the host material.

### 3.2. AFM Analysis

**Figure 3:**
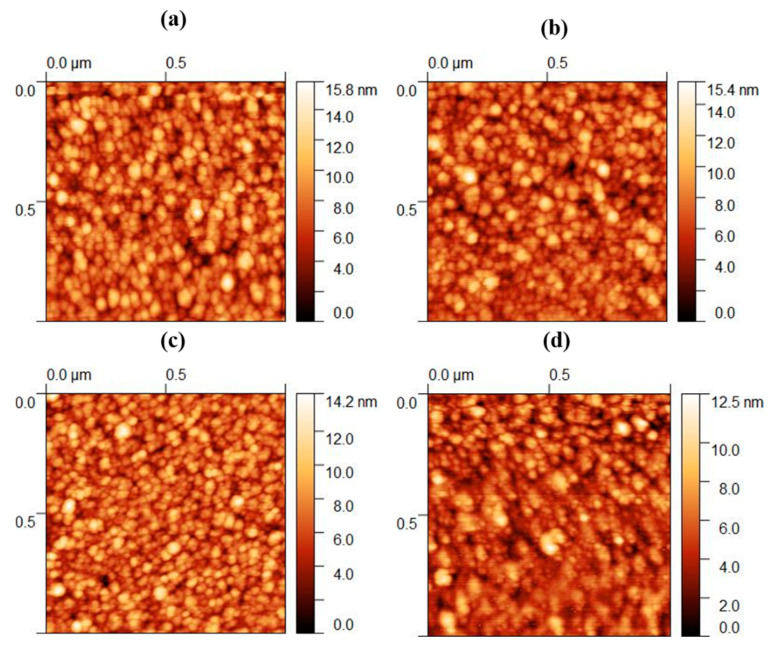
AFM micrographs of ZnO doped with TM. (**a**) Pure, (**b**) Co, (**c**) Fe, and (**d**) Zr.

The analysis of the AFM micrographs of the TM-doped ZnO thin films reveals a distinct feature: the presence of smaller grain sizes compared to pure ZnO (see Figure 3). This phenomenon could potentially stem from the fusion of individual grains. This intricate surface morphology showcases densely packed grains. The significant reduction in the grain size becomes particularly evident in the ZnO thin films doped with Co, Fe, and Zr. This shrinking grain size is likely attributed to nucleation on the surface, fostering the formation of smaller grains within the larger ones. The introduction of TM doping plays a pivotal role in creating nucleation sites and heightening interfacial energy, thereby impeding further crystal growth. To counterbalance this effect, particle clustering intervenes, diminishing the interfacial energy and leading to a decrease in the average grain size, as observed. Another important observation is the increase in pores and voids within thin films. This could be attributed to the incorporation of sputtering gas (argon) during the deposition process, potentially coupled with an escalation in the number of vacancies. Furthermore, the decrease in the AFM-measured grain size aligns coherently with an increase in the crystallinity observed in the TM-doped ITO thin films, mirroring the reduction in the XRD-derived crystallite size. The average grain size of ZnO, Co: ZnO, Fe: ZnO, and Zr: ZnO from the AFM analysis was calculated as 57.47 nm, 52.38 nm, 48.21 nm, and 45.62 nm, respectively.

### 3.3. Elemental Analysis

**Figure 4:**
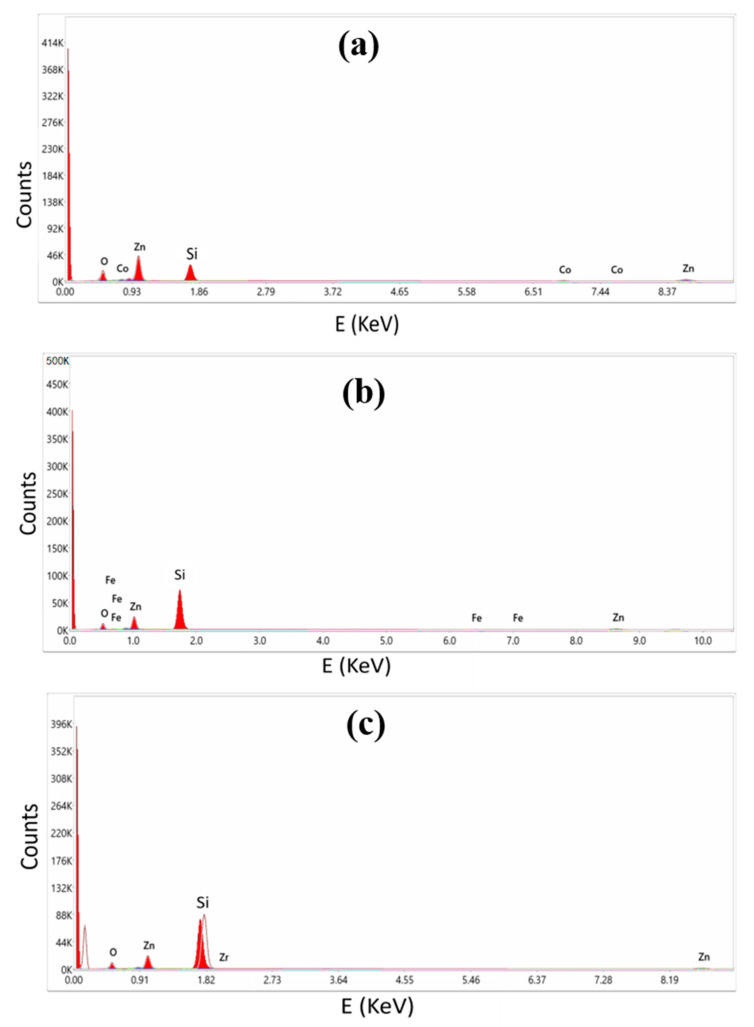
EDX Spectra of (**a**) Co: ZnO, (**b**) Fe: ZnO, and (**c**) Zr: ZnO.

The EDX spectroscopy of the deposited thin films is shown in Figure 4. Different elements emit X-rays at varying energies, and this variation is often reflected in the position or intensity of a peak in the energy-dispersive X-ray (EDX) spectrum. Therefore, the peak or highest energy level in an EDX spectrum may be used as a distinguishing feature for element identification. O makes up 57.49% of the composition, Zn makes up 39.79%, and Co contributes 2.72%, according to the analysis of the weight percentages of the Co: ZnO thin film. The elemental make-up of the Fe: ZnO thin film was calculated to be 61.72 percent oxygen, 37.16 percent zinc, and 1.12 percent iron. The study of the Zr: ZnO thin film, on the other hand, showed that its constituent elements were 62.4% oxygen, 37.49% zinc, and 0.11% zirconium. The number of atoms of a certain element that have been excited by the incident beam may be inferred from the intensity of the peak in the radiation spectrum. Some peaks in the EDX spectrum can arise from the substrate or support material, particularly when it is not fully transparent to X-rays or contains elements capable of generating characteristic X-ray emissions. The substrate’s influence on the spectrum is notable when it scatters X-rays, induces fluorescence from its elements upon X-ray excitation, or contributes to backscattering. To mitigate these effects, it is common to employ thin samples or opt for X-ray transparent substrates, and background subtraction techniques may be applied to correct substrate-related contributions during analysis.

### 3.4. FTIR Analysis

**Figure 5:**
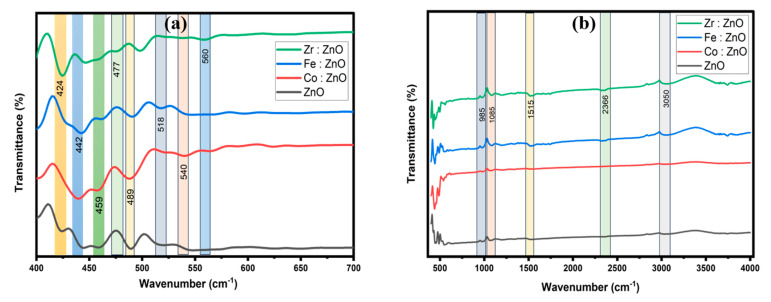
FTIR spectra of ZnO and TM-doped ZnO thin films ranging from (**a**) 400 to 700 cm^−1^, (**b**) 400 to 4000 cm^−1^.

The FTIR studies confirmed the deposition of ZnO wurtzite structures in the TM-doped ZnO samples. As indicated in Figure 5b, similar spectra were obtained for both the undoped ZnO and the TM-doped ZnO samples. All samples are scanned in the 400–4000 cm^−1^ range using the transmittance mode. The FTIR spectra were analyzed using the findings of nanostructures presented in the literature [43,44,45]. The predominant absorption peaks in the 400–700 cm^−1^ region for all the doped and undoped ZnO samples might be ascribed to ZnO stretching modes [46]. The detected absorption peaks in our FTIR spectra were in the range of 900 to 1100 cm^−1^, matching to C-O-C stretching vibration and related to the oxygen stretching and bending frequency [47]. The measured peak in the 1100–1600 cm^−1^ region corresponds to the Zn-OH bending mode [48] as well as the C-OH plane bending and C-OH out-of-plane bending [49]. The detected absorption peaks in the 2200 to 2400 cm^−1^ range match CO_2_ stretching vibration. Overlapping O-H and C-H stretching modes can describe a band in the 2900–3700 cm^−1^ range. C-H local vibrational modes with frequencies ranging from 2800 to 3100 cm^−1^ have been found in a few semiconductors [50,51,52]. The symmetric and antisymmetric C-H stretching modes are given to the local vibrational modes in these materials. Upon closer inspection, discernible alterations in the peak intensity and width became evident following the introduction of Co, Fe, and Zr. These changes strongly imply the impact of these elements on the ZnO lattice. As illustrated in Figure 5a, the inclusion of these rare earth metals seemed to prompt a noticeable shift in the associated peaks. This shift implies modifications in the bond length and molecular mass, indicating the interaction between the transition metals and ZnO.

### 3.5. Optical Analysis

**Figure 6:**
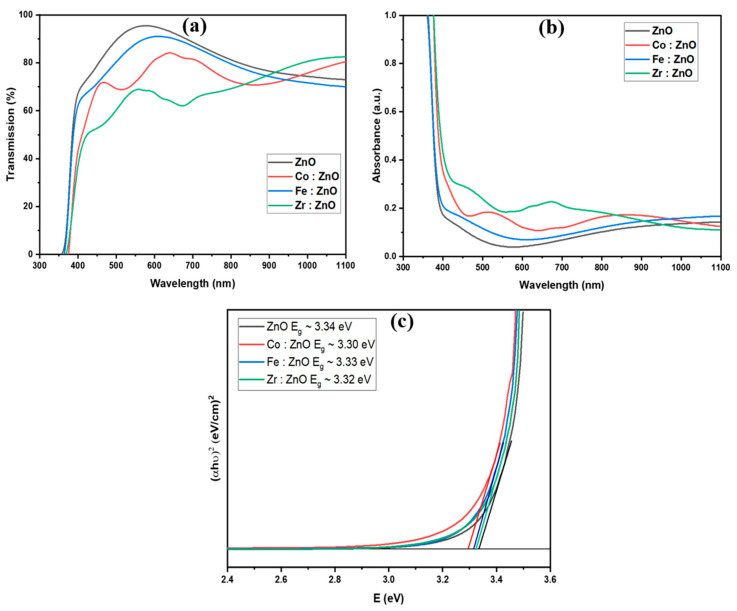
(**a**) Transmission, (**b**) absorption Spectra, and (**c**) bandgap of ZnO and TM-doped ZnO thin films.

The deposited thin films’ optical properties were analyzed using a UV spectrophotometer with a wavelength range of 320 to 1200 nm. Figure 6a,b display the transmission and absorption spectra of the ZnO and TM-doped ZnO thin films. The transmittance measurements show relatively low values in the UV area but high values in the visible and near-infrared. We have also confirmed that the incorporation of transition metals into ZnO does not change the material’s intrinsic absorption edge. The absorption edge has been shown to move to longer wavelengths. At a wavelength of around 590 nm, the thin films of ZnO, Fe: ZnO, Co: ZnO, and Zr: ZnO showed maximum transmittances of roughly 98%, 90%, 84%, and 68%, respectively.

The optical bandgap was evaluated using Tauc plots. The graphs represented in Figure 6c show the proportionality between the square of the absorption coefficient (*α*) times the energy of a photon (*hv*) and the energy of a photon (eV). The following mathematical equation was used to calculate the bandgap energy *E_g_* [53]:

(7) αhυ=Ahυ−Eg12

*E_g_* = 1242/λ (8)

λ = Wavelength(9)

Linear extrapolation is used in Figure 6c to provide the optical bandgap values for the ZnO and TM-doped ZnO thin films. The as-deposited ZnO thin film has a bandgap measured at 3.34 eV. The optical bandgap was found to decrease somewhat from 3.34 to 3.30 eV when transition metal doping was introduced. Transition metal incorporation into the ZnO layer is thought to be responsible for the observed shift. Overlapping wave functions between the electrons and impurity atoms may lead to energy level localization and band-tailing effects, which may explain the observed narrowing of the bandgap [54,55].

### 3.6. PL Analysis

The ZnO and TM-doped ZnO thin films’ PL properties were studied using a spectrofluorometer, model FS5, excited by a xenon arc lamp. Validating electronic transitions in semiconductor materials with thin film architectures through photoluminescence is a common practice. Two main sources of PL emissions in ZnO have been identified in the literature [56]. The first is related to deep-level emissions, whereas the second is thought to be due to near-band edge excitonic (NBE) processes. Typically caused by excitonic or band–band recombination, NBE emission occurs at the absorption edge of a material. When dopants, native defects, or complexes are present, bound excitons are thought of as irrelevant transitions. Different electronic states may be generated by these entities inside the bandgap [57].

Deep-level emissions are the emissions that can be seen by the naked eye (from 400 to 750 nm) [56]. Enhancements in electron transmission inside the bandgap caused by various crystallographic or surface imperfections account for the emission peaks seen in the visible spectrum. Doping causes the creation of new defect states that lie below the conduction band. In turn, this leads to certain excited electrons in the conduction band relaxing to these defect states [58,59]. Defects in the ZnO structure may be divided into two classes: extrinsic and intrinsic. Oxygen vacancy, zinc vacancy, oxygen interstitial, zinc interstitial, oxygen anti-site, and zinc anti-site are all intrinsic or native values. These flaws may coexist in clusters with other intrinsic faults and with exogenous components, and they can take on a variety of charged states or be neutral [57,60].

**Figure 7:**
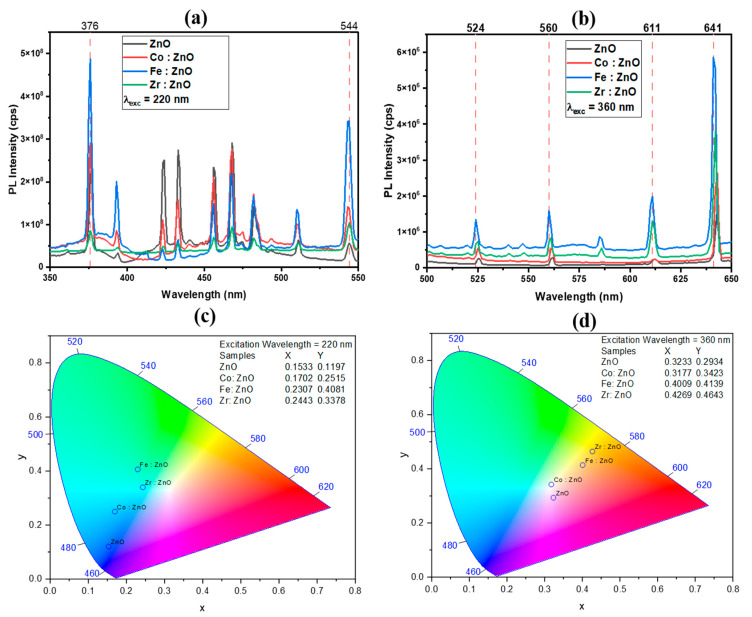
PL (**a**,**b**) and chromaticity (**c**,**d**) of ZnO and TM-doped ZnO thin films.

Figure 7a,b display the photoluminescence (PL) spectra of the thin films formed at two distinct excitation wavelengths (220 nm and 360 nm). The emission peaks may be seen in the ultraviolet, indigo, blue, and green spectrum when excited at 220 nm. In general, the UV emissions from the NBE radiative transition have their first significant peak at 376 nm; the recombination of free excitons generates this emission through an exciton–exciton collision mechanism with an absorption edge at 370 nm, and it serves to validate the bandgap of the deposited thin films [61,62]. The conduction band to zinc vacancy (or oxygen antisite) transition causes emissions from 420 to 490 nm. The green band’s inception may be traced to emissions at wavelengths between 490 and 550 nm. The green band originates from an oxygen vacancy to interstitial zinc atom and zinc vacancy to shallow donor state transitions, as well as zinc vacancy to zinc vacancy transitions. The emission peaks are in the green, yellow, and red spectrum, at an excitation wavelength of 360 nm. The green spectral line might be caused by radiation with a wavelength between 500 and 570 nm. Transitions between deep donor oxygen vacancies and interstitial Zn atoms, as well as transitions from shallow donor states to zinc vacancies, are responsible for the green band. The yellow and orange band may be the result of emission between 570 and 620 nm. The yellow emission band is thought to be caused by Oi flaws. As energy is transferred from the conduction band to the levels produced inside the bandgap, a yellowish light is emitted. This change in the energy level between Zni (Zinc Interstitial) and Oi (Oxygen Interstitial) is mirrored in the orange emission phenomena. The red spectrum may result from radiation with a wavelength of 620–650 nm. Zinc vacancy complexes or zinc atoms may cause red emission bands in interstices [63,64]. It is well-known that transition metal dopants introduce new energy levels inside the bandgap of ZnO. Inside the bandgap, defects may be created at the energy levels, which are often associated with the d orbitals of the dopant. Enhanced radiative recombination processes may result from electrons transitioning between these states, which in turn contributes to the amplification of the PL intensity [65]. The slight variation in peak intensity and peak broadness can be demonstrated in Figure 6a,b. The specific shift observed in the photoluminescence peaks upon doping depends on the type and concentration of the dopants, the host material, and the local environment created by the dopant atoms. Doping introduces impurity atoms into the crystal lattice, creating new energy levels within the bandgap of the host material. These energy levels can act as recombination centers for electrons and holes, affecting the emission and absorption of photons and leading to a shift in photoluminescence peaks. Doping can alter the band structure of the material by introducing new energy bands or modifying existing ones. This modification influences the electronic transitions responsible for photoluminescence, causing a shift in the emission or absorption spectra.

The optical analysis reveals that Fe: ZnO exhibits a wider bandgap and high transparency. This unique property results in enhanced and intense emission in the visible spectrum when excited at different wavelengths (i.e., 220 nm and 360 nm). Consequently, Fe: ZnO emerges as a promising alternative to conventional ZnO in photoluminescence and photovoltaic applications. Furthermore, the increased electrical conductivity observed in Fe: ZnO opens up potential applications in LED technology and photovoltaic systems. The combination of a wider bandgap, high transparency, and enhanced electrical conductivity positions Fe: ZnO as a versatile material with promising prospects for advancement in LED and photovoltaic applications.

Figure 7c,d are chromaticity diagrams that show how the use of deposited thin films provides a wide range of colors, representing the visible spectrum. According to the following figure, there is potential for the material under study to be used in optoelectronic devices. The chromaticity refers to the variety of colors possible, including all permutations of the primary colors (blue, green, and red) and all shades in between. The properties of hue and saturation are graphically represented on a two-dimensional X–Y plane. The chromaticity diagram maps colors in a two-dimensional space using color coordinates. Commonly used color spaces for this purpose include the CIE 1931 xy chromaticity diagram. The diagram represents colors using two coordinates (x, y), allowing for the visualization of the entire color spectrum. Photoluminescence involves the emission of light, and the resulting photoluminescent spectrum provides information about the distribution of emitted light at different wavelengths. By converting this spectral information into color coordinates on the chromaticity diagram, one can understand the perceived color of the emitted light.

The middle section of the picture shows how white light may be created by mixing different colors at different intensities to produce the 460 nm (blue), 520 nm (green), and 680 nm (red) of the visible spectrum. We can easily estimate the proximity of the emitted light to white light, an aspect vividly depicted in Figure 7c,d. To accurately forecast the outcome of the additive mixing process involving two or more colored lights, the chromaticity diagram is used.

### 3.7. Electrical Analysis

**Figure 8:**
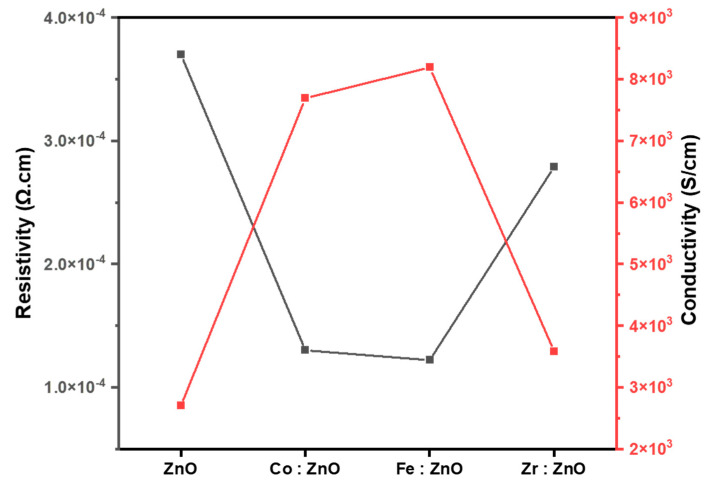
Electrical analysis of ZnO and TM–doped ZnO thin films.

The conductivity and electric resistivity of thin films of ZnO and TM (Co, Fe, and Zr)-doped ZnO are shown in Figure 8. Doping Co into ZnO enhances the carrier concentration and improves the charge carrier transit, as seen in the figure, leading to higher conductivity and lower resistivity. In addition, Fe doping in ZnO increases the conductivity, whereas Zr doping increases the carrier concentration. Zr doping raises the fermi level, and moves the fermi level into the band, all of which reduce the diffusion length of the carriers and, in turn, decreases the conductivity. ZnO’s bandgap is widened due to the incorporation of impurity energy levels by the transition metal dopants. These energy levels may generate new charge carriers by absorbing or re-emitting electrons from the valence band [66,67].

### 3.8. Application in Solar Cell

Transparent conducting oxides (TCOs) represent a class of materials renowned for their dual functionality of transparency and electrical conductivity. The hallmark of TCOs lies in their high electrical conductivity, facilitating efficient electricity conduction—a crucial attribute for their integration into electronic devices. Simultaneously, TCO exhibits transparency to visible light, allowing for the unhindered passage of light without significant absorption or scattering. This transparency is pivotal in applications such as the development of transparent conductive films [68,69,70]. Typically possessing a wide bandgap, TCOs ensure transparency to visible light, a prerequisite for maintaining optical clarity.

In the course of our research, we made noteworthy observations regarding the performance of Fe-doped ZnO thin films in comparison to other Zr-doped ZnO and Co-doped ZnO thin films. The Fe-doped ZnO showcased superior electrical conductivity, positioning it as an efficient conductor of electricity. Furthermore, the Fe-doped ZnO exhibited heightened transparency and a broader bandgap when compared to the Zr-doped ZnO and Co-doped ZnO. It is noteworthy that the Fe-doped ZnO demonstrated a significant enhancement in the n-type electrical conductivity over the pure ZnO thin films.

Of particular interest is the distinct potential of Fe-doped ZnO as a transparent electrode in various applications, particularly in the realm of solar cells. The combination of its impressive electrical conductivity and enhanced transparency, coupled with the widened bandgap, positions Fe-doped ZnO as a promising candidate for deployment as a transparent electrode. Beyond its role as a transparent electrode, Fe-doped ZnO holds great promise as an electron transport layer in solar cell configurations, where its unique combination of optical and electrical characteristics could contribute to an enhanced overall performance.

## 4. Conclusions

The successful deposition of thin films comprising ZnO and transition metal (Co, Fe, and Zr)-doped ZnO was achieved through a combined RF and DC co-sputtering process. The structural analysis revealed the persistence of the wurtzite structure, characteristic of ZnO, in the thin films post-doping with transition metals. Doping induced changes in the lattice characteristics, indicating the incorporation of dopant ions into the ZnO lattice. The detailed examination of morphology through AFM provided valuable insights into the effects of transition metal doping. The presence of cobalt (Co), iron (Fe), and zirconium (Zr) in the doped ZnO thin films was confirmed by employing energy-dispersive X-ray spectroscopy (EDS). Chemical bonding investigations underscored strong bonds between the dopant components and the ZnO matrix, influencing the electrical structure of the thin films. The optical properties of the doped zinc oxide thin films underwent significant alterations due to the introduction of cobalt, iron, and zirconium dopants. Notably, there was a distinct broadening of the absorption edge, consistent with a narrowing of the bandgap in the thin films. The introduction of dopants had a substantial impact on the optical transparency of the materials, ranging from 98% to 68%. These outcomes suggest the potential applicability of these materials in various optoelectronic areas. The photoluminescence (PL) spectroscopy revealed multiple emission peaks within the visible spectrum, attributed to the direct and indirect recombination of electrons and holes. The electrical characterization investigations demonstrated that the presence of dopants significantly influenced the conductivity of the thin films.

## Figures and Tables

**Table 1:** Detailed structural parameters of deposited thin films.

Parameters	ZnO	Co: ZnO	Fe: ZnO	Zr: ZnO
Crystalline size (Dp)	49.26	47.58	44.83	43.71
Microstrain (ε)	2.951	3.5911	3.709	4.022
Dislocation density (δ)	0.412	0.4416	0.497	0.523
Lattice parameter (a)	3.006	3.004	3.003	3.003
Lattice parameter (c)	5.169	5.151	5.145	5.122
Volume (V)	23.0148	23.0148	22.998	22.9208
Bond length (L)	1.850	1.850	1.847	1.847
Internal relaxation parameter (u)	0.2943	0.2951	0.2971	0.2971
Specific surface area (S)	21.73	21.84	23.87	24.49

## Data Availability

Data are contained within the article.
